# Isochromophilones from an endophytic fungus *Diaporthe* sp.

**DOI:** 10.1007/s13659-012-0023-2

**Published:** 2012-04-13

**Authors:** Le-Yun Zang, Wei Wei, Ting Wang, Ye Guo, Ren-Xiang Tan, Hui-Ming Ge

**Affiliations:** Institute of Functional Biomolecules, State Key Laboratory of Pharmaceutical Biotechnology, School of Life Science, Nanjing University, Nanjing, 210093 China

**Keywords:** azaphilone, endophyte, *Diaporthe* sp., cytotoxicity

## Abstract

Three new azaphilone compounds, isochromophilones X–XII (**1–3**), together with two known ones sclerotioramine (**4**) and isochromophilone VI (**5**) were isolated from the cultures of an endophytic fungus *Diaporthe* sp. The structures were elucidated by extensive HRESIMS and NMR spectroscopic analyses. All compounds were tested for their cytotoxicities against five human cancer cell lines by MTT method, among which compound **1** showed moderate inhibitory effects on these cell lines. This was the first report of azaphilones isolated from *Diaporthe* sp.

**Electronic Supplementary Material:**

Supplementary material is available for this article at 10.1007/s13659-012-0023-2 and is accessible for authorized users.

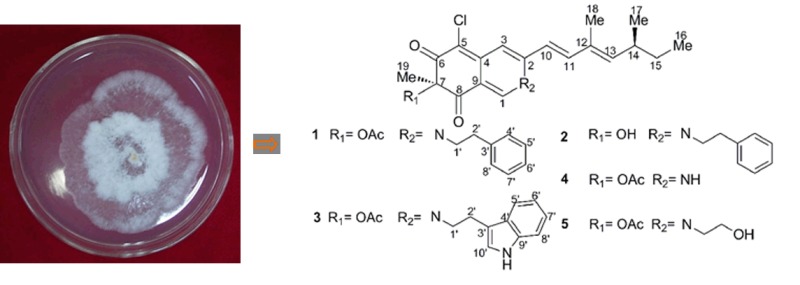

## Introduction

The azaphilones are a structurally diverse family of natural products containing a highly oxygenated, bicyclic core and a chiral quaternary center. The name of azaphilone arose as a result of their affinity for ammonia: the pigments react with amines, such as proteins, amino acids and nucleic acids, to form red and purple vinylogous $\gamma$-pyridones due to the exchange of pyrane oxygen for nitrogen[1–3]. Azaphilone compounds had a wide range of bioactivities including cytotoxic, antifungal, antimicrobial, antioxidant and anti-inflammatory activities,[4–8] and could be generally produced by *Penicillium*, *Chaetomium* and *Monascus*.[3] In the course of our search for novel and/or bioactive metabolites from microbes lived in special niche,[4,9,10] five azaphilones including three new isochromophilones X–XII (**1**–**3**) and two known compounds sclerotioramine (**4**)[11] and isochromophilone VI (**5**)[12] were isolated from the solid-substrate fermentation cultures of an endophytic fungus *Diaporthe* sp. The structures were identified on the basis of extensive spectroscopic methods. The cytotoxic activities of compounds (**1**–**5**) against human gastric cancer SGC-7901, colorectal carcinoma SW1116, breast adenocarcinoma MCF-7, lung adenocarcinoma epithelial A549 and melanoma A375 cells were evalutated by MTT method. Compound **1** exhibited moderate cytotoxic activities with its $IC_{50}$ values ranging from 14.90–35.75 $\mu$M.

## Results and Discussion

Compound **1** was obtained as red needles, the molecular formula was assigned as $C_{29}H_{32}ClNO_4$ evidenced by its HRESIMS spectrum at *m/z* 494.2080 $[M + H]^+$ (calcd. for

**Figure 1**. Compounds **1**–**5** from *Diaporthe* sp.

$C_{29}H_{33}{}^{35}ClNO_4$, 494.2098), indicating 14 degrees of unsaturation. Compound **1** displayed similar $^1H$ NMR spectrum to that of known compound isochromophilone VI (**5**) except for a monosubstituted benzene ring signals [$\delta$ 7.26 (m, 1H), 7.32 (m, 2H), 7.10 (d, 7.0, 2H)] in **1**, which suggested that the 2'-OH in isochromophilone VI (**5**) could be replaced by a benzene ring in compound **1**. To confirm the proposed structure, extensive NMR spectra have been investigated (Figure 2). $^1H$-$^1H$ COSY correlations of H-10/H-11, and of Me-18/H-13/H-14/H-15(Me-17)/Me-16, together with the HMBC correlations from H-10 to C-12, H-11 to C-18, H-13 to C-11, C-15, C-17, C-18 indicated the presence of a 3,5-dimethyl-1,3-heptadiene residue. On the basis of the coupling constants of H-10, H-11, and the NOE correlation of H-11 with H-13, the configurations of these two double bonds were both determined to be *E*. The HMBC correlations from H-1' to

*To whom correspondence should be addressed. E-mail: hmge@nju.edu.cn (H.M. Ge); rxtan@nju.edu.cn (R.X. Tan).

**Table 1.** **$^1$H (500MHz) and $^{13}$C NMR (125MHz) data for 1–3 (in $CDCl_3$)**

| | **1** | | **2** | | **3** | |
|---|---|---|---|---|---|---|
| No. | $\delta_H$ (mult., *J*) | $\delta_C$ | $\delta_H$ (mult., *J*) | $\delta_C$ | $\delta_H$ (mult., *J*) | $\delta_C$ |
| 1 | 7.53 (s) | 140.8, CH | 7.49 (s) | 140.0, CH | 7.49 (s) | 141.0, CH |
| 2 | | 147.8, C | | 148.3, C | | 148.3, C |
| 3 | 6.96 (s) | 111.5, CH | 6.96 (s) | 111.0, CH | 6.94 (s) | 111.4, CH |
| 4 | | 144.3, C | | 145.7, C | | 144.6, C |
| 5 | | 103.1, C | | 100.0, C | | 102.1, C |
| 6 | | 184.3, C | | 187.6, C | | 207.3, C |
| 7 | | 84.8, C | | 83.4, C | | 84.8, C |
| 8 | | 193.7, C | | 196.3, C | | 193.8, C |
| 9 | | 114.6, C | | 115.5, C | | 114.6, C |
| 10 | 5.98 (d, 15.5) | 114.6, CH | 6.02 (d, 15.5) | 114.4, CH | 5.85 (d, 15.0) | 114.8, CH |
| 11 | 6.90 (d, 15.5) | 144.9, CH | 6.94 (d, 15.5) | 145.3, CH | 6.84 (d, 15.0) | 144.9, CH |
| 12 | | 131.5, C | | 131.5, C | | 131.7, C |
| 13 | 5.68 (d, 9.5) | 148.0, CH | 5.71 (d, 10.0) | 148.4, CH | 5.63 (d, 9.5) | 147.7, CH |
| 14 | 2.47 (m) | 35.0, CH | 2.49 (m) | 35.1, CH | 2.43 (m) | 35.0, CH |
| 15 | 1.44 (m); 1.35 (m) | 30.0, $CH_2$ | 1.44 (m); 1.34 (m) | 30.0, $CH_2$ | 1.42 (m); 1.33 (m) | 30.1, $CH_2$ |
| 16 | 0.89 (t, 7.5) | 11.9, $CH_3$ | 0.89 (t, 7.5) | 11.9, $CH_3$ | 0.87 (t, 7.5) | 12.0, $CH_3$ |
| 17 | 1.03 (d, 6.5) | 20.2, $CH_3$ | 1.03 (d, 6.6) | 20.2, $CH_3$ | 1.00 (d, 6.5) | 20.2, $CH_3$ |
| 18 | 1.81 (d, 1.0) | 12.6, $CH_3$ | 1.83 (s) | 12.6, $CH_3$ | 1.56 (s) | 12.1, $CH_3$ |
| 19 | 1.52 (s) | 23.2, $CH_3$ | 1.51 (s) | 29.5, $CH_3$ | 1.48 (s) | 23.2, $CH_3$ |
| 7-OAc | 2.16 (s) | 20.3, $CH_3$ | | | 2.17 (s) | 20.3, $CH_3$ |
| | | 170.0, C | | | | 170.1, C |
| 1' | 4.13 (m); 3.98 (m) | 55.4, $CH_2$ | 4.17 (m); 4.03 (m) | 55.6, $CH_2$ | 4.11 (m) | 54.5, $CH_2$ |
| 2' | 3.02 (m) | 36.6, $CH_2$ | 3.05 (m) | 36.4, $CH_2$ | 3.20 (m) | 26.5, $CH_2$ |
| 3' | | 135.8, C | | 135.6, C | | 109.6, C |
| 4' | 7.10 (d, 7.0) | 128.7, CH | 7.08 (d, 7.0) | 128.7, CH | | 126.7, C |
| 5' | 7.32 (m) | 129.2, CH | 7.32 (m) | 129.3, CH | 7.48 (d, 8.0) | 117.7, CH |
| 6' | 7.26 (m) | 127.7, CH | 7.26 (m) | 127.8, CH | 7.14 (t, 8.0) | 120.1, CH |
| 7' | 7.32 (m) | 129.2, CH | 7.32 (m) | 129.3, CH | 7.22 (t, 8.0) | 122.7, CH |
| 8' | 7.10 (d, 7.0) | 128.7, CH | 7.08 (d, 7.0) | 128.7, CH | 7.37 (d, 8.0) | 111.8, CH |
| 9' | | | | | | 136.5, C |
| 10' | | | | | 6.94 (s) | 123.2, CH |

C-3', H-4' to C-2', H-7' to C-3' confirmed the linkage of C-2' and the benzene ring. Moreover, the HMBC correlations of H-1 to C-1', C-2, C-4, C-5, C-8, C-9, of H-3 to C-5 and C-10, of Me-19 to C-6, C-8 indicated the presence of isoquinoline-6,8(2*H*,7*H*)-dione moiety, a typical group in azaphilone skeleton and the way the residues linked at. Thus, the planar structure of **1** was established.

Compound **2**, isolated as red needles, was determined to have the molecular formula $C_{27}H_{30}ClNO_3$ (13 degrees of unsaturation) based on an HRESIMS peak at *m/z* 452.1998 $[M + H]^+$ (calcd. for $C_{27}H_{31}{}^{35}ClNO_3$, 452.1992), 42 amu ($C_2H_2O$) less than compound **1**. Its $^1$H NMR spectrum revealed nearly all identical structural features to those found in compound **1**, except the absence of an acetyl signal in the $^1$H NMR spectrum (Table 1) and thus the structure of compound **2** could be readily determined as shown in Figure 1 which was supported by 2D NMR spectra.

Compound **3** was established the molecular formula as $C_{31}H_{33}ClN_2O_4$ by HRESIMS analysis (*m/z* 533.2203, calcd. for $C_{31}H_{34}{}^{35}ClN_2O_4$, 533.2207) with 16 degrees of unsaturation. The $^1$H NMR spectroscopic data of compound **3** were well comparable to those of **1**. Considering the molecular formula, an indole ring might substitute 2'-OH of isochromophilone VI in the compound **3**. The HMBC (Figure 2) correlations from H-10' to C-4' and C-9', from H-5' to C-7' and C-9', from H-8' to C-4' and C-6' confirmed the presence of indole ring.

The 7*R*-configurations of isochromophilones X–XII (**1**–**3**) were determined by circular-dichroism (CD) spectroscopy. The observed positive *Cotton* effect at the wavelength (370–390 nm) was well comparable to that of 7*R*-5-chloro-7,8-dihydro-3-(3-hydroxypropyl)-7-methyl-6,8-dioxo-6*H*-2-benzopyran-7-yl ester.[13,14] Furthermore, the 14*S*-configurations of **1**–**3** were determined by comparing their NMR data of the side chain moieties with those of similar azaphilone compounds, isochromophilone Ia, IIa and rotiorinol A.[15,16]

**Figure 2**. Key $^1$H-$^1$H COSY (bold), HMBC (arrow) correlations of **1** and **3**

Biosynthetically, azaphilone was derived from the polyketide pathway,[3,17] whose oxygen atom could be further substituted by primary amines or ammonia into *N*-containing compounds rapidly.[1] For example, sclerotiorin could be converted to sclerotioramine (**4**) with ammonia.[2] Thus, compounds **1** and **3** were deduced as consequences of sclerotiorin reacting with phenylethanamine and tryptamine, the decarboxylated product of phenylalanine and tryptophan, respectively (Scheme 1). The further deacetylation of compound **1** gave compound **2**. Also, we can deduce that compounds **1**–**3** possessed the same configurations as that of sclerotiorin (Figure 1) by their biosynthetic view.

**Scheme 1.** Proposed biogenetic pathway for isochromophilones X–XII (**1**–**3**)

All compounds were evaluated for their cytotoxicities against MCF-7, SGC-7901, SW1116, A549 and A375 cell lines by MTT method. Only, isochromophilone X (**1**) showed moderate cytotoxic activities with its $IC_{50}$ values of 14.90, 16.84, 24.15, 26.93 and 35.75 $\mu$M against MCF-7, SGC-7901, SW1116, A549 and A375 cell lines (Table 2), respectively, while the other compounds exhibited no activities with $IC_{50}$ values greater than 50 $\mu$M.

## Experimental Section

**General Experimental Procedures**. HRESIMS spectra were recorded on an Agilent 6210 TOF LC/MS. CD spectra were obtained on a JASCO J-810 spectrometer. IR spectra were obtained on a Nexus 870 FT-IR spectrometer. NMR spectra were recorded on Bruker AV-500, AV-300 or DRX500 NMR spectrometer. Optical rotations were recorded on a Rudolph Autopol III automatic polarimeter. UV spectra were recorded on a Hitachi U-3000 spectrophotometer. Silica gel (200–300 mesh; Qingdao Marine Chemical Factory, Qingdao, China) and Sephadex LH-20 gel (Pharmacia Biotech, Sweden) were used for column chromatography (CC). HPLC was performed with a Hitachi L-7110 pump, and UV detector L-7400 equipped with an Apollo C18 column (5 $\mu$m, 250 mm × 4.6 mm; Alltech Associates, Inc. Chicago, IL, USA).

**The Fungal Material and Cultivation Conditions.** The strain IFB-3lp-10 was isolated from the healthy leaves of *Rhizophora stylosa* collected in August 2010 from the mangrove forest of Hainan Province of China. The strain was identified as *Diaporthe* sp. by comparing the morphological character and 18S rDNA sequence with that of a standard record. A voucher specimen has been deposited in the Institute of Functional Biomolecules, State Key Laboratory of Pharmaceutical Biotechnology, Nanjing University. After growing on PDA medium at 28 °C for 5 d, the fungus *Diaporthe* sp. IFB-3lp-10 was inoculated into Erlenmeyer flasks (1000 mL) containing 400 mL of ME liquid medium. After incubation for 4 d at 28 °C on a rotary shaker at 150 rpm, 20 mL of culture liquid was transferred as the seed into 250 mL flasks, each preloaded with the evenly mingled medium (7.5 g of grain, 7.5 g of bran, 0.5 g of yeast extract, 0.1 g of sodium tartrate, 0.01 g of $FeSO_4{\cdot}7H_2O$, 0.1 g of sodium glutamate and 30 ml of $H_2O$). The fungus then grew for 30 d at 28 °C with the relative humidity in the range 60–70%.

**Extraction and Isolation.** The air-dried samples (30 kg) were extracted with 95% EtOH and the organic solvent was evaporated to dryness under a vacuum to afford a crude extract (1.2 kg) which gave six fractions (Fr.1, 21g; Fr.2, 75 g; Fr.3, 56 g; Fr.4, 25 g; Fr.5, 36 g; Fr.6, 158 g) upon column chromatography (10 × 120 cm) on silica gel (6000 g, 200–300 mesh) eluted with a gradient of $CH_2Cl_2$/MeOH (v/v 100:0, 100:1, 100:2, 100:4, 100:8, 100:20, 0:100, each 20L) based on TLC monitoring. Purification of the second subfraction ($CH_2Cl_2$/MeOH 100:1) by using column chromatography (petroleum ether/EtOAc 1:1) and Sephadex LH-20 (100% MeOH) followed by HLPC (90% MeOH) gave **1** (15.0 mg) and **2** (2.5 mg). Purification of the third subfraction ($CH_2Cl_2$/MeOH 100:2) in the same way mentioned above gave **3** (3.6 mg). The fourth fraction was subjected to reversed phase ODS column (4 cm × 40 cm) with a gradient of MeOH/$H_2O$ (v/v 20:80, 30:70, 40:60, 50:50, 60:40, 70:30, 80:20, 90:10, 100:0, each 3 L) to give nine subfractions, respectively. Purification of the fifth subfraction (MeOH/$H_2O$ 60:40) by using column chromatography (petroleum ether/EtOAc 1:2) and Sephadex LH-20 (100 % MeOH) gave **4** (2.8 g) and **5** (64 mg).

**Isochromophilones X (1):** red needles. $[\alpha]_D^{20} = +1116.4$ ($c$ = 0.028, MeOH). UV/Vis (MeOH): $\lambda_{max}$/nm (log $\varepsilon$) = 201 (4.5), 227 (4.4), 373 (4.5). CD ($c$ 2.8 × $10^{-4}$ g $ml^{-1}$, MeOH) $\lambda_{max}$/nm ($\Delta\varepsilon$): 229.5 (+0.4), 246 (+6.2), 304 (−8.9), 381.5 (+7.9), 429 (+1.2). IR (KBr): $\nu_{max}$ = 3490.4, 2960.7, 2924.7, 1701.8, 1594.6, 1501.6, 1262.5, 1220.3, 1144.3, 1081.7, 1032.0, 801.9 $cm^{-1}$. HRESIMS: *m/z* 494.2080 ($[M + H]^+$, calcd. for $C_{29}H_{33}{}^{35}ClNO_4$, 494.2098). For $^1H$ and $^{13}C$ NMR spectroscopic data, see Table 1.

**Isochromophilones XI (2):** red needles. $[\alpha]_D^{20} = +596.0$ ($c$ = 0.08, MeOH). UV/ Vis (MeOH): $\lambda_{max}$/nm (log $\varepsilon$) = 206 (4.5), 228 (4.5), 350 (4.5). CD ($c$ 8.0 × $10^{-4}$ g $ml^{-1}$, MeOH) $\lambda_{max}$/nm ($\Delta\varepsilon$): 209 (+5.6), 236 (+0.6), 249 (+1.4), 311 (−4.3), 375 (+3.6), 414.5 (+0.5). IR (KBr): $\nu_{max}$ = 3419.2, 2962.5, 1588.8, 1494.7, 1261.5, 1096.2, 1033.4, 801.5 $cm^{-1}$. HRESIMS: *m/z* 452.1998 ($[M + H]^+$, calcd. for $C_{27}H_{31}{}^{35}ClNO_3$, 452.1992). For $^1H$ and $^{13}C$ NMR spectroscopic data, see Table 1.

**Isochromophilones XII (3):** red needles. $[\alpha]_D^{20} = +936.0$ ($c$ = 0.025, MeOH). UV/ Vis (MeOH): $\lambda_{max}$/nm (log $\varepsilon$) = 201 (4.6), 221 (4.7), 375 (4.3). CD ($c$ 2.5 × $10^{-4}$ g $ml^{-1}$, MeOH) $\lambda_{max}$/nm ($\Delta\varepsilon$): 229.5 (−1.6), 246.5 (+2.4), 304.5 (−4.5), 379

**Table 2.** ***In vitro*** **cytotoxicity of compound 1**

| $IC_{50}$ ($\mu$M) | MCF-7 | SGC-7901 | SW1116 | A549 | A375 |
|---|---|---|---|---|---|
| **1** | 14.90 ± 2.41 | 16.84 ± 1.06 | 24.15 ± 1.77 | 26.93 ± 1.17 | 35.75 ± 2.11 |
| Doxorubicin•HCl[a] | 0.55 ± 0.03 | 2.03 ± 0.05 | 5.09 ± 1.13 | 3.37 ± 1.66 | 1.75 ± 0.24 |

[a]used as a positive control

(+3.5), 419.5 (+0.3). IR (KBr): $\nu_{max}$ = 3440.3, 3237.5, 2958.9, 1736.0, 1574.3, 1486.1, 1219.3, 1145.0, 758.9 $cm^{-1}$. HRESIMS: *m/z* 533.2203 ($[M + H]^+$, calcd. for $C_{31}H_{34}{}^{35}ClN_2O_4$, 533.2207). For $^1H$ and $^{13}C$ NMR spectroscopic data, see Table 1.

**Cytotoxicity Assay.** The cytotoxic effects of **1**–**5** on the viability of the SGC-7901, SW1116, MCF-7, A549 and A375 cell lines were assayed with the MTT [3-(4,5-dimethylthiazol-2-yl)-2,5-diphenyltetrazolium bromide] colorimetric method. The data represent the mean of three experiments performed in triplicate and are expressed as means ± SD.

## Electronic Supplementary Material

Supplementary material is available in the online version of this article at http://dx.doi.org/ 10.1007/s13659-012-0023-2 and is accessible for authorized users.

## Acknowledgments

This work was co-financed by National Natural Science Foundation of China (81121062, 21132004, 90813036, and 81172948), MOE (NCET-10-0477), and Jiangsu Provincial Government (BK2009010).

**Open Access** This article is distributed under the terms of the Creative Commons Attribution License which permits any use, distribution, and reproduction in any medium, provided the original author(s) and source are credited.

## Electronic supplementary material

Supplementary material, approximately 819 KB.
