# Supplementary material for: Isochromophilones from an endophytic fungus Diaporthe sp
Source: Nat Prod Bioprospect. 2012 Apr 13;2(3):117–20. doi: 10.1007/s13659-012-0023-2 (PMC4131592; doi:10.1007/s13659-012-0023-2)

Electronic Supplementary Material

Isochromophilones from an endophytic fungus *Diaporthe* sp.

Le-Yun ZANG, Wei WEI, Ting WANG, Ye GUO, Ren-Xiang TAN,* and Hui-Ming GE*

Institute of Functional Biomolecules, State Key Laboratory of Pharmaceutical Biotechnology, School of Life Science, Nanjing University, Nanjing 210093, China

Received 15 March 2012; Accepted 27 March 2012

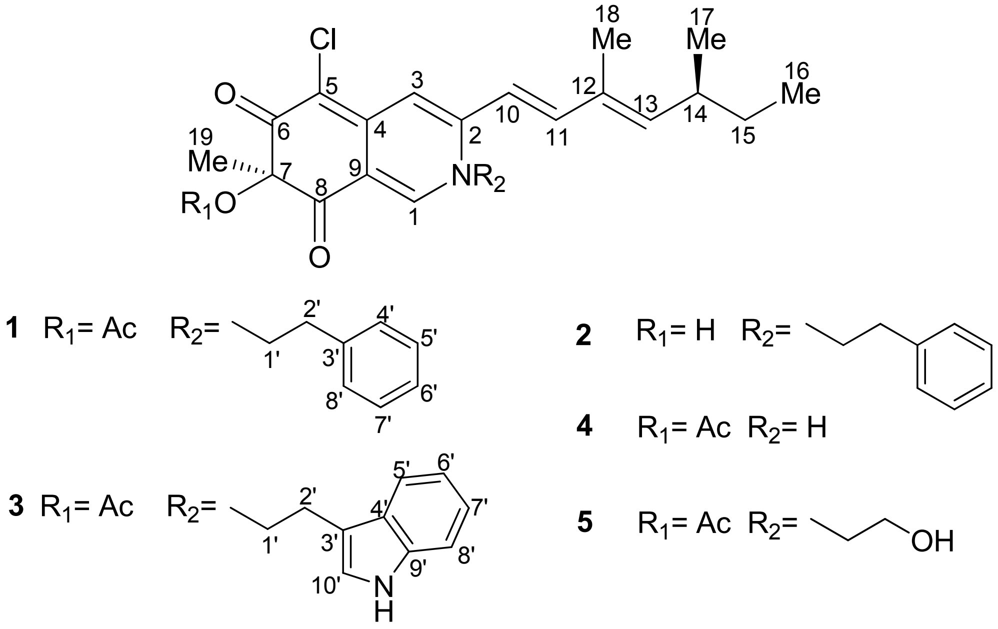

Structure of compounds **1**–**5**

*To whom correspondence should be addressed. E-mail: hmge@nju.edu.cn (H.M. Ge); rxtan@nju.edu.cn (R.X. Tan).

Content list:

S1. ^{1}H NMR spectrum of isochromophilone X (**1**) in $CDCl_3$ (500 MHz).
S2. ^{13}C NMR spectrum of isochromophilone X (**1**) in $CDCl_3$ (125 MHz).
S3. DEPT spectrum of isochromophilone X (**1**) in $CDCl_3$ (125 MHz).
S4. HSQC spectrum of isochromophilone X (**1**) in $CDCl_3$ (500 MHz).
S5. ^{1}H-^{1}H COSY spectrum of isochromophilone X (**1**) in $CDCl_3$ (500 MHz).
S6. HMBC spectrum of isochromophilone X (**1**) in $CDCl_3$ (500 MHz).
S7. ROESY spectrum of isochromophilone X (**1**) in $CDCl_3$ (500 MHz).
S8. CD spectrum of isochromophilone X (**1**) in MeOH.
S9. ^{1}H NMR spectrum of isochromophilone XI (**2**) in $CDCl_3$ (500 MHz).
S10. ^{13}C NMR spectrum of isochromophilone XI (**2**) in $CDCl_3$ (125 MHz).
S11. DEPT spectrum of isochromophilone XI (**2**) in $CDCl_3$ (125 MHz).
S12. HSQC spectrum of isochromophilone XI (**2**) in $CDCl_3$ (500 MHz).
S13. ^{1}H-^{1}H COSY spectrum of isochromophilone XI (**2**) in $CDCl_3$ (500 MHz).
S14. HMBC spectrum of isochromophilone XI (**2**) in $CDCl_3$ (500 MHz).
S15. CD spectrum of isochromophilone XI (**2**) in MeOH.
S16. ^{1}H NMR spectrum of isochromophilone XII (**3**) in $CDCl_3$ (500 MHz).
S17. ^{13}C NMR spectrum of isochromophilone XII (**3**) in $CDCl_3$ (125 MHz).
S18. HSQC spectrum of isochromophilone XII (**3**) in $CDCl_3$ (300 MHz).
S19. ^{1}H-^{1}H COSY spectrum of isochromophilone XII (**3**) in $CDCl_3$ (500 MHz).
S20. HMBC spectrum of isochromophilone XII (**3**) in $CDCl_3$ (300 MHz).
S21. CD spectrum of isochromophilone XII (**3**) in MeOH.

S1. ^{1}H NMR spectrum of isochromophilone X (1) in $CDCl_3$ (500 MHz)

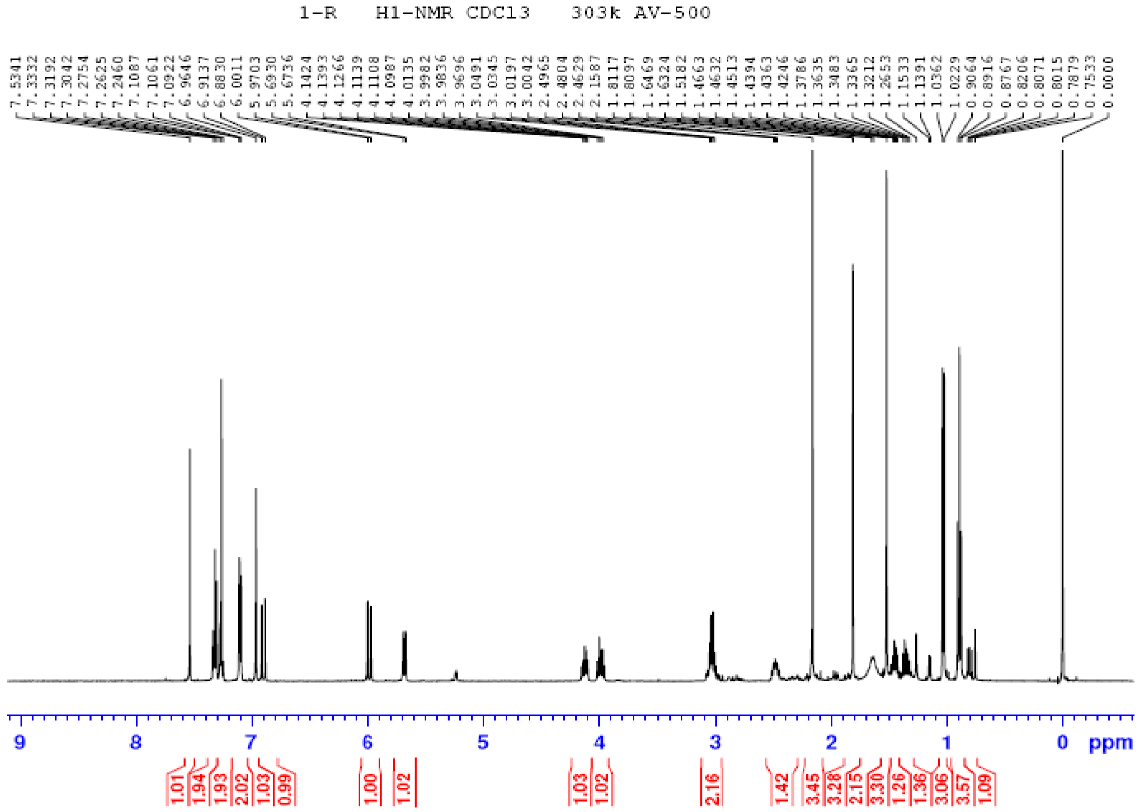

S2. ^{13}C NMR spectrum of isochromophilone X (1) in $CDCl_3$ (125 MHz).

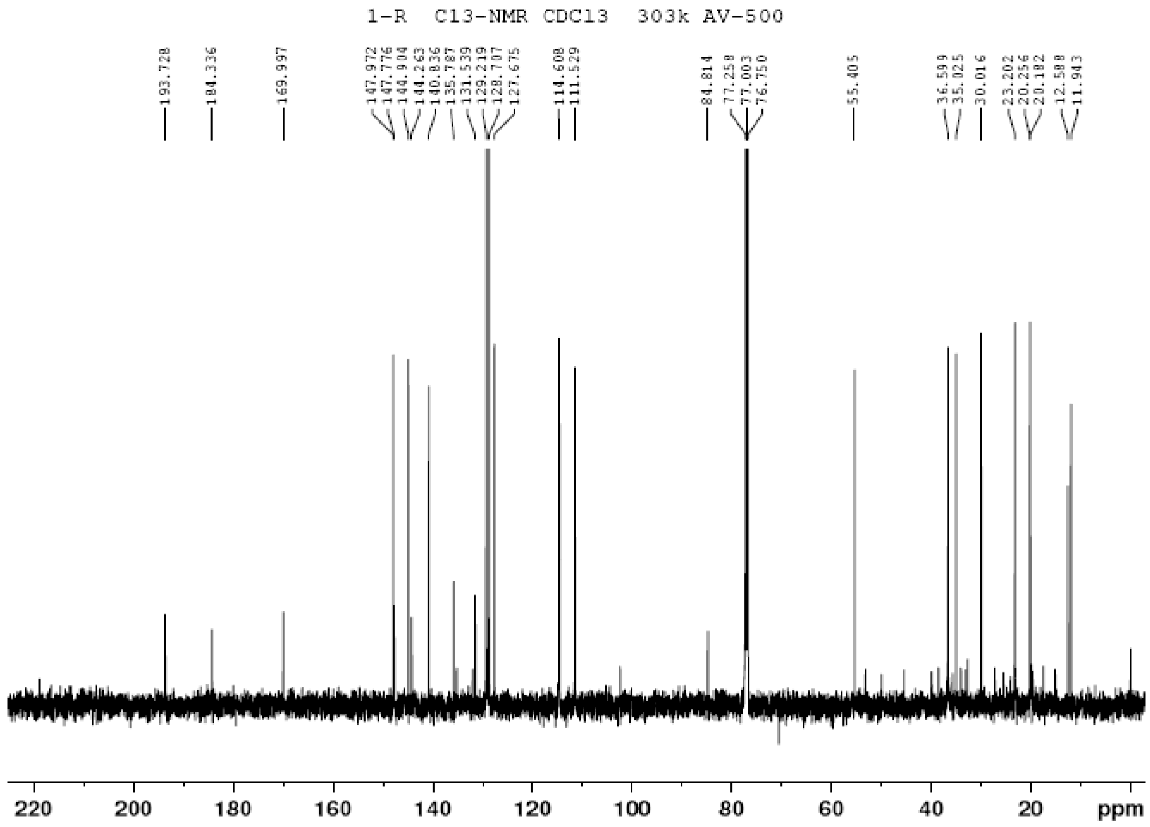

S3. DEPT spectrum of isochromophilone X (1) in $CDCl_3$ (125 MHz).

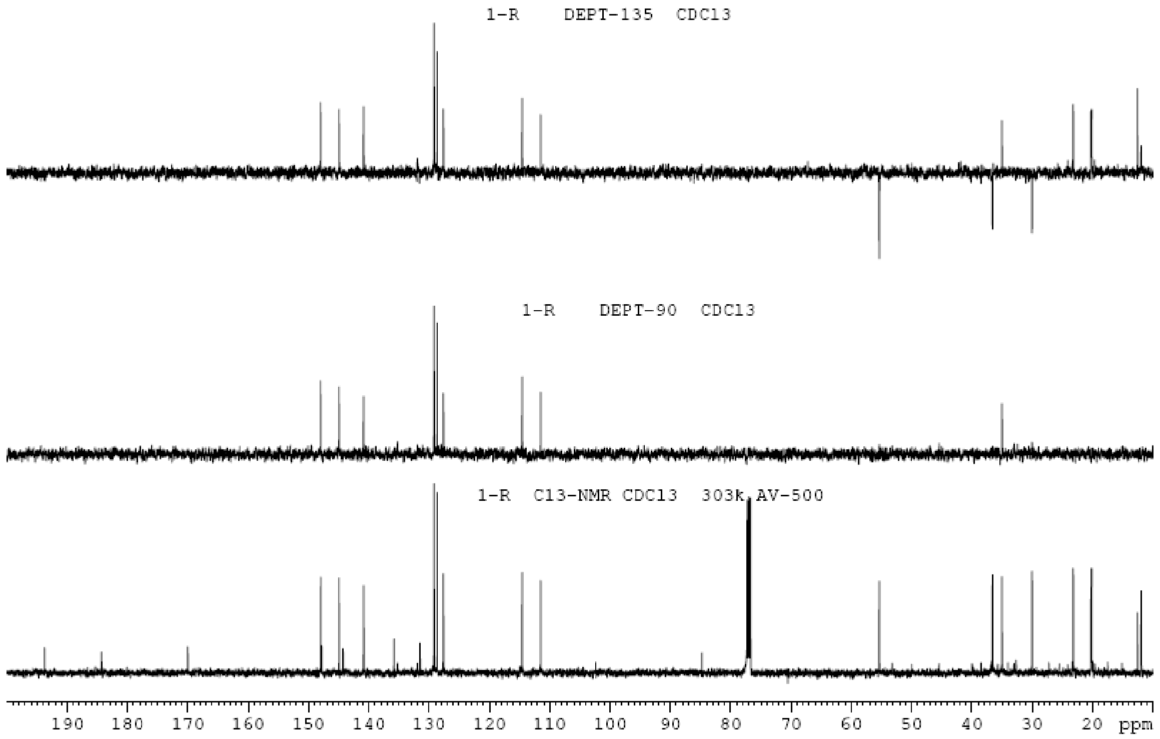

S4. HSQC spectrum of isochromophilone X (1) in $CDCl_3$ (500 MHz).

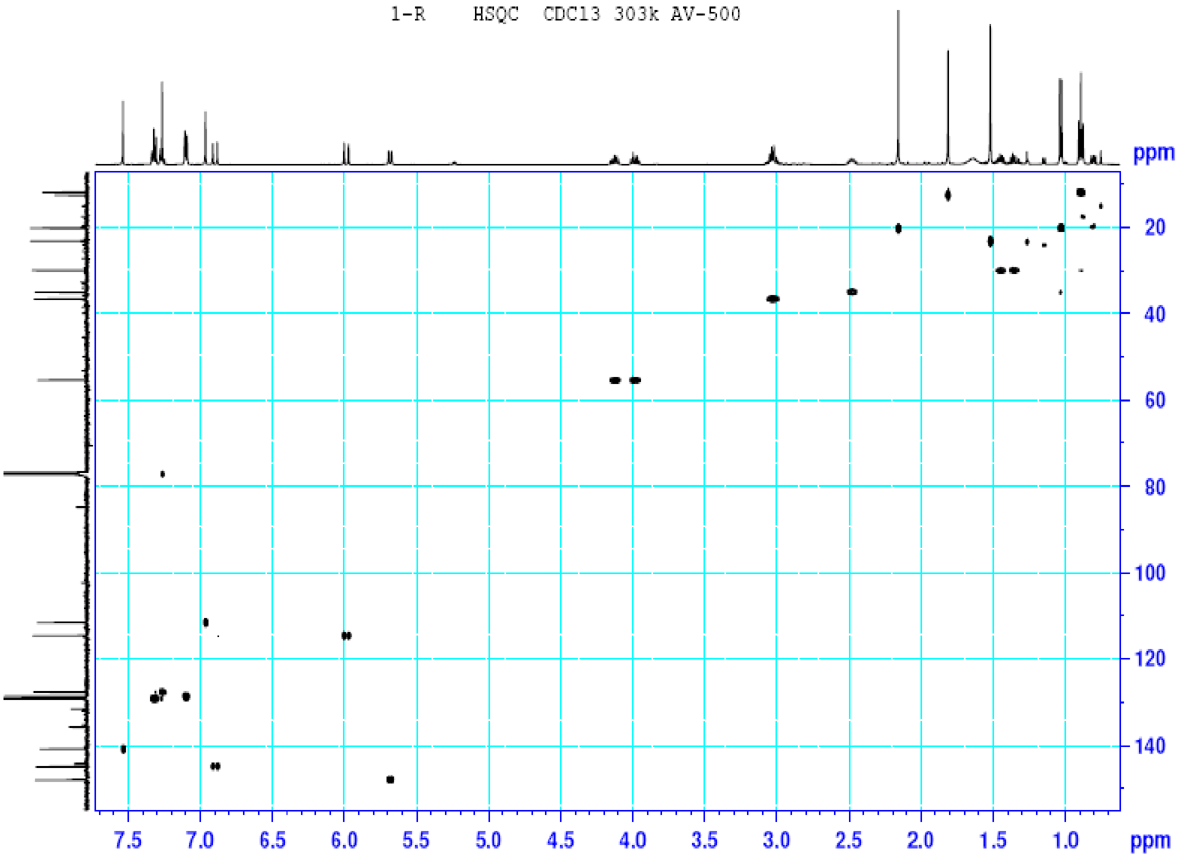

S5. ^{1}H-^{1}H COSY spectrum of isochromophilone X (1) in $CDCl_3$ (500 MHz).

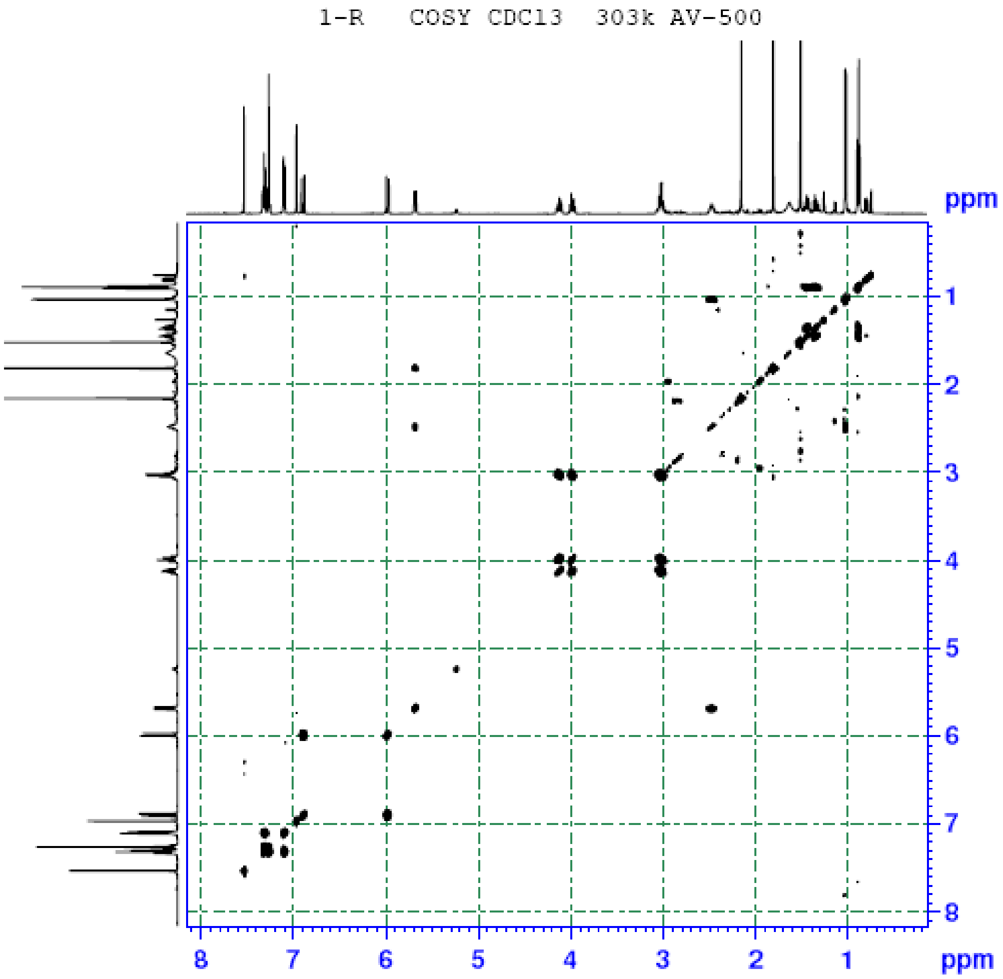

S6. HMBC spectrum of isochromophilone X (1) in $CDCl_3$ (500 MHz).

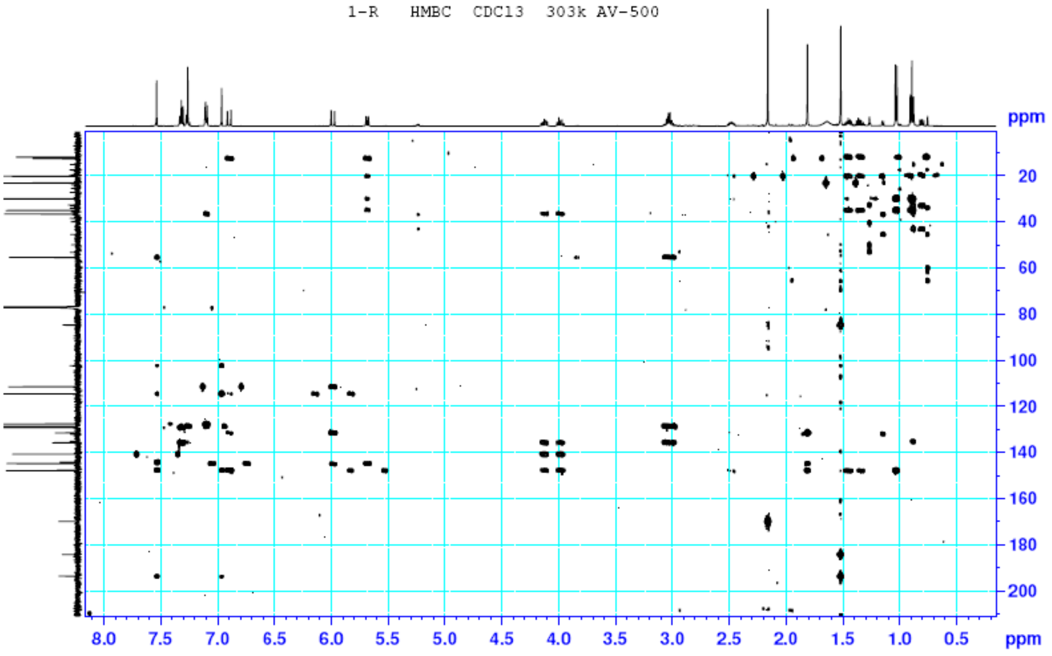

S7. ROESY spectrum of isochromophilone X (1) in $CDCl_3$ (500 MHz).

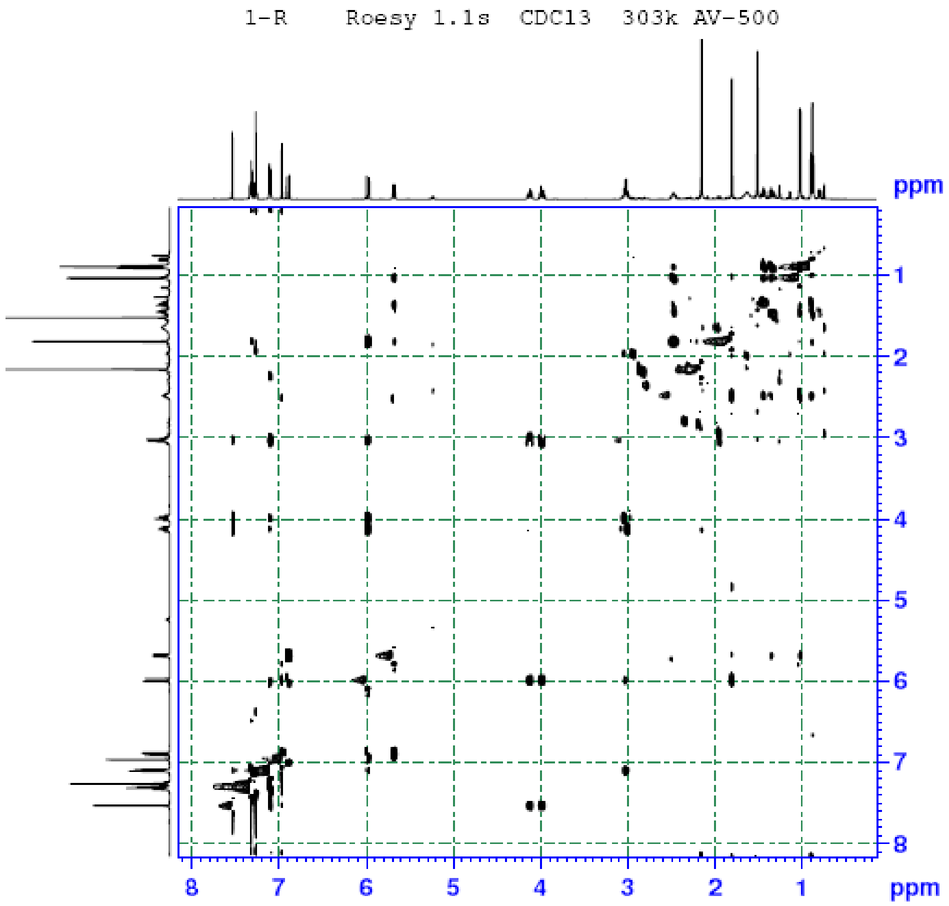

S8. CD spectrum of isochromophilone X (1) in MeOH

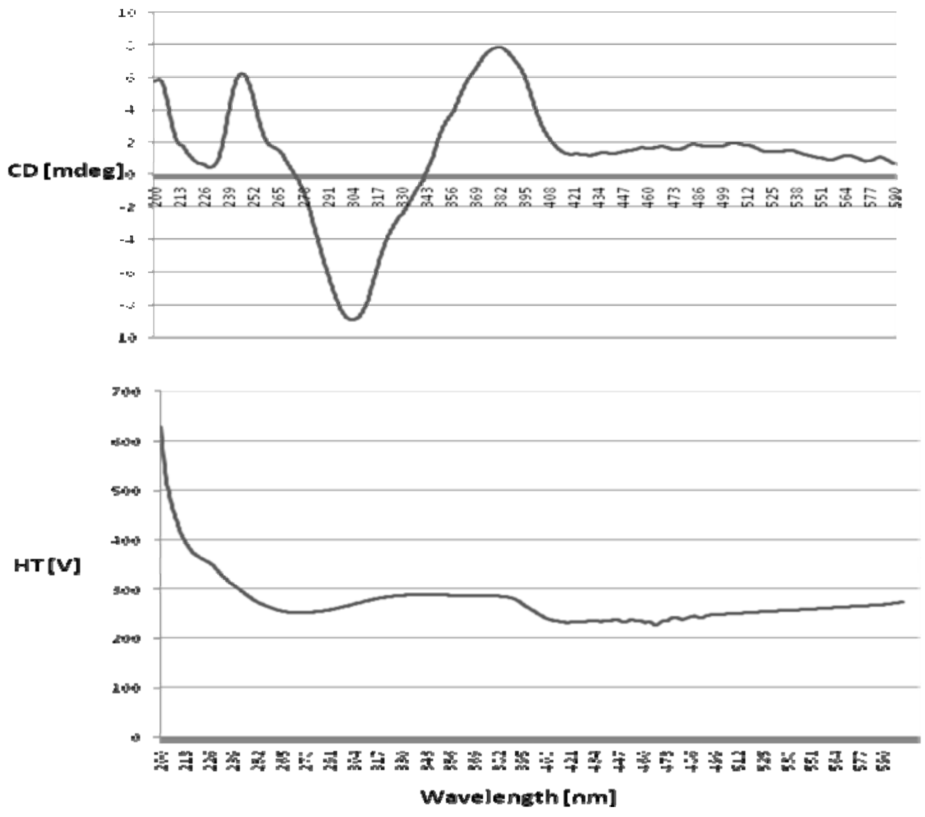

S9. ^{1}H NMR spectrum of isochromophilone XI (2) in $CDCl_3$ (500 MHz).

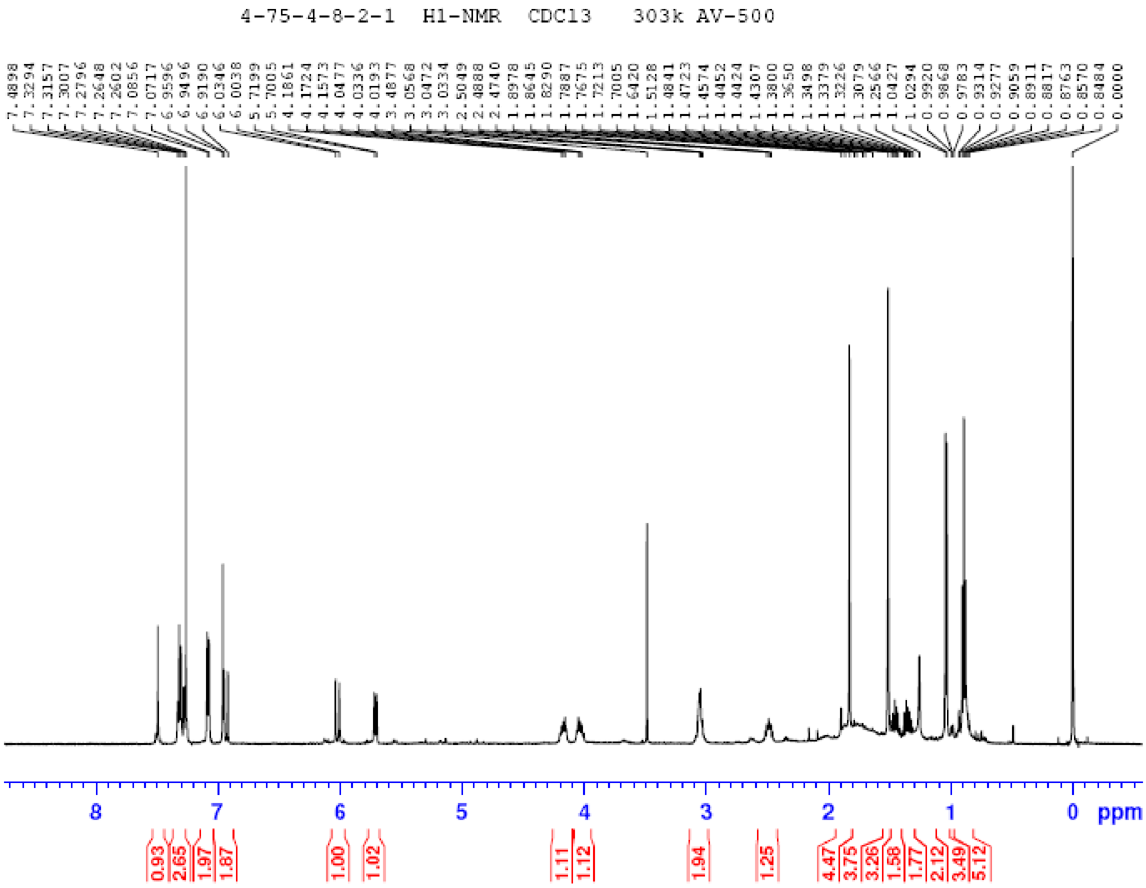

S10. ^{13}C NMR spectrum of isochromophilone XI (2) in $CDCl_3$ (125 MHz).

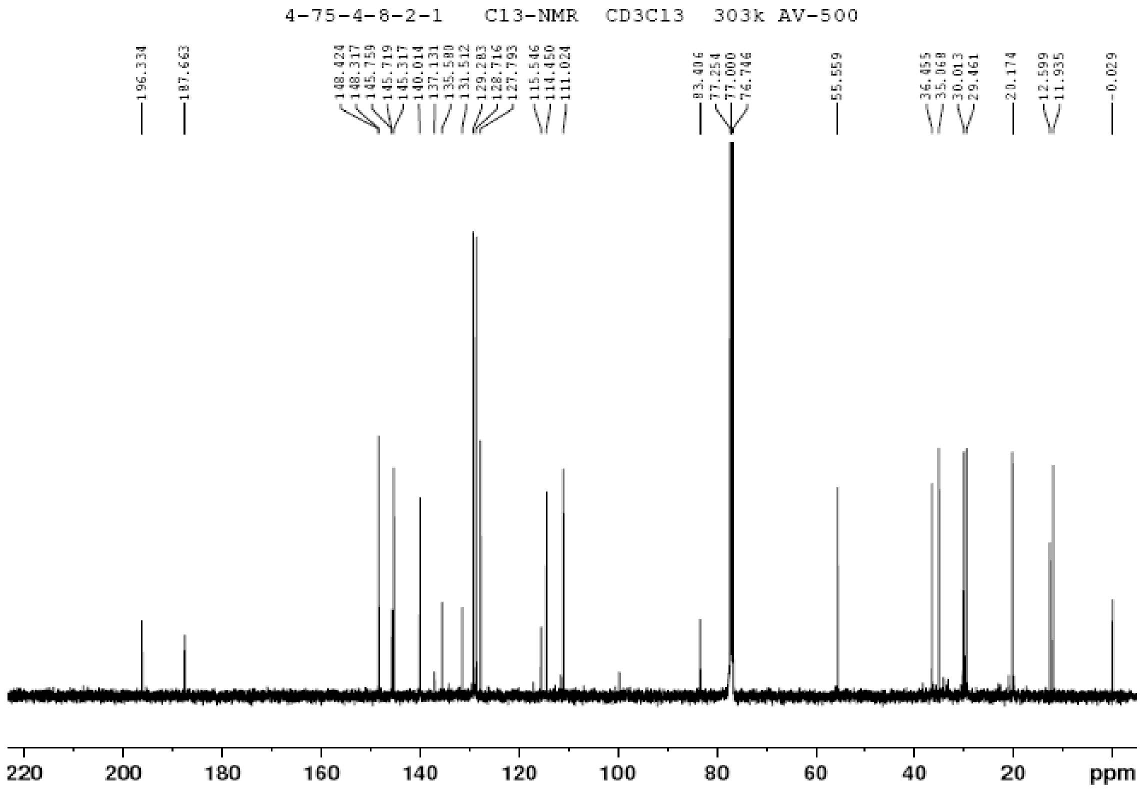

S11. DEPT spectrum of isochromophilone XI (2) in CDCl$_3$ (125 MHz).

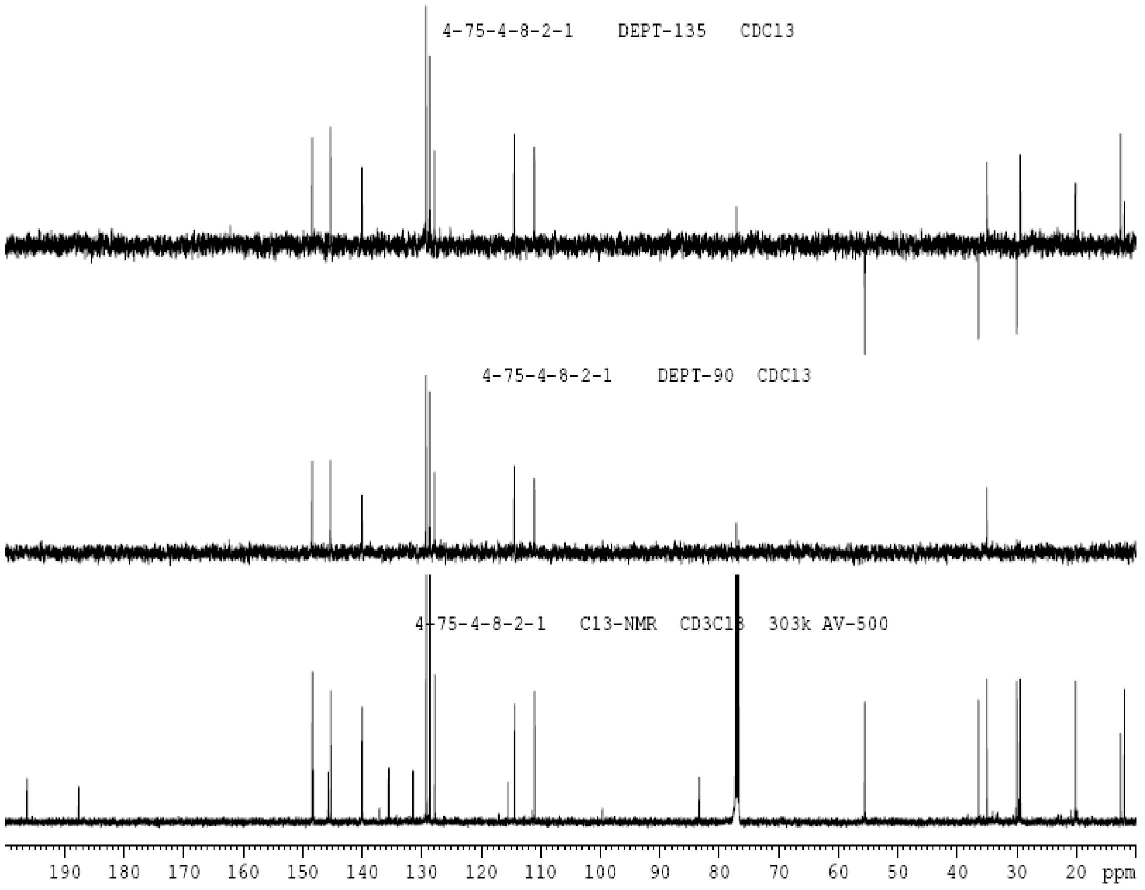

S12. HSQC spectrum of isochromophilone XI (2) in CDCl$_3$ (500 MHz).

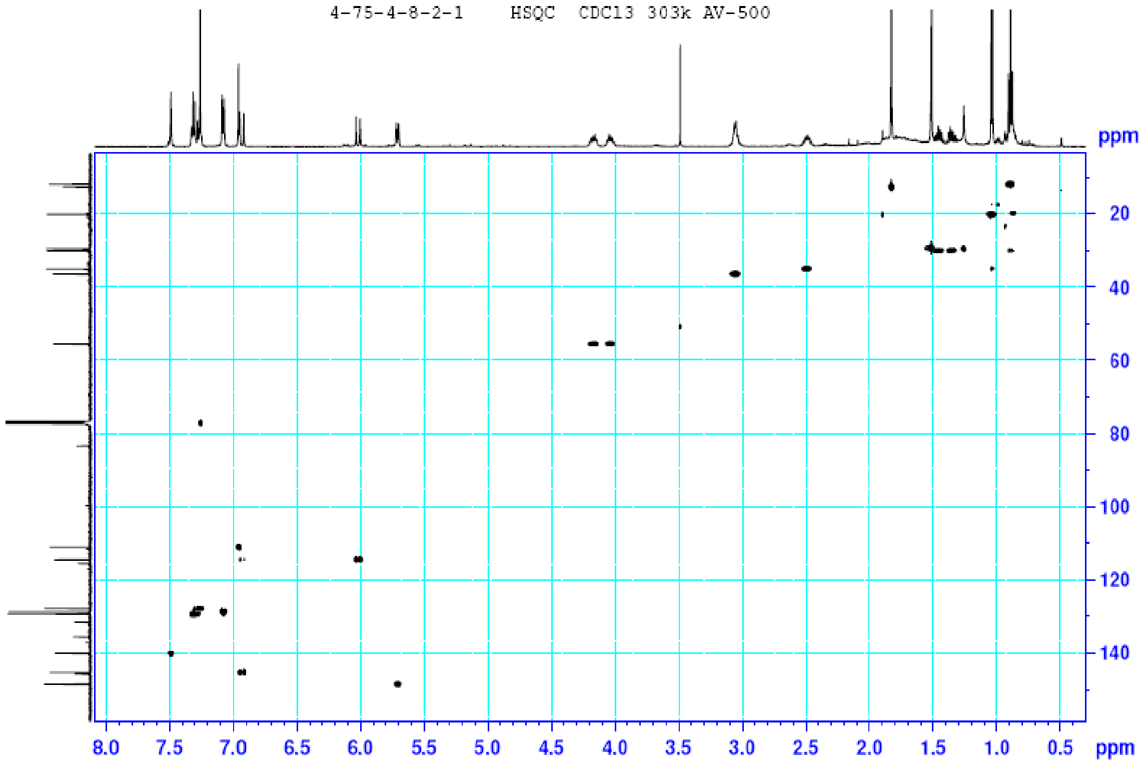

S13. ^{1}H-^{1}H COSY spectrum of isochromophilone XI (2) in $CDCl_3$ (500 MHz).

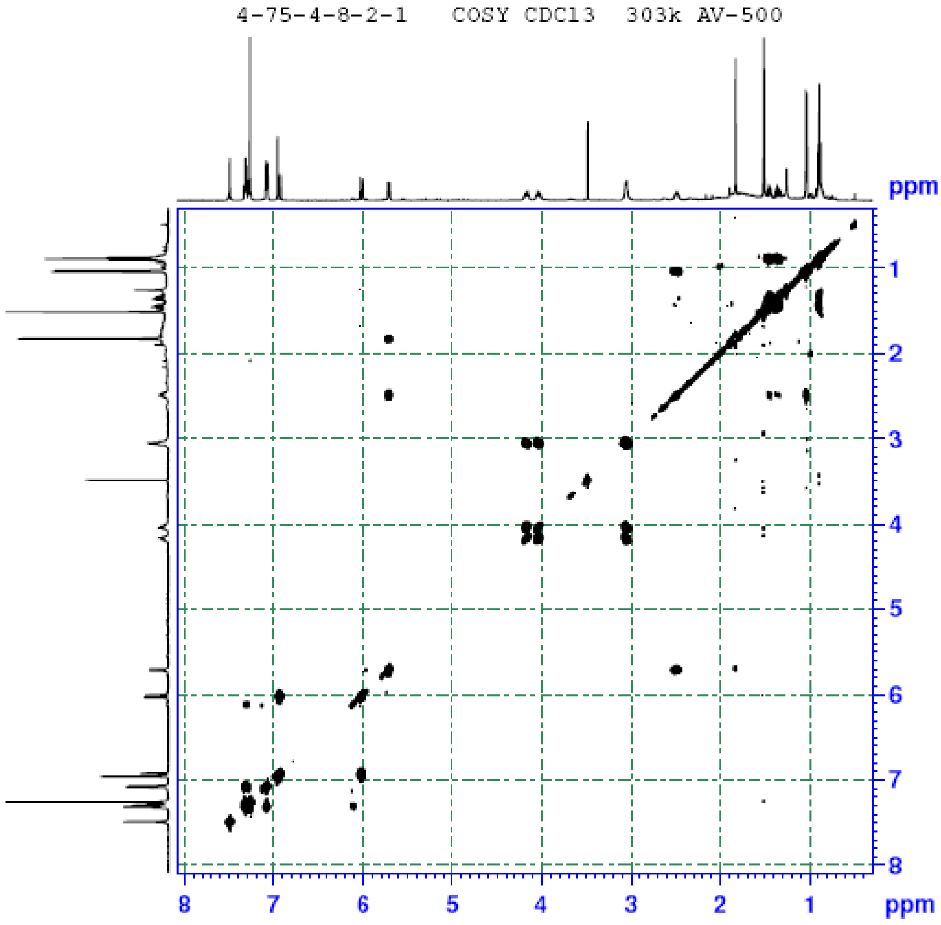

S14. HMBC spectrum of isochromophilone XI (2) in $CDCl_3$ (500 MHz).

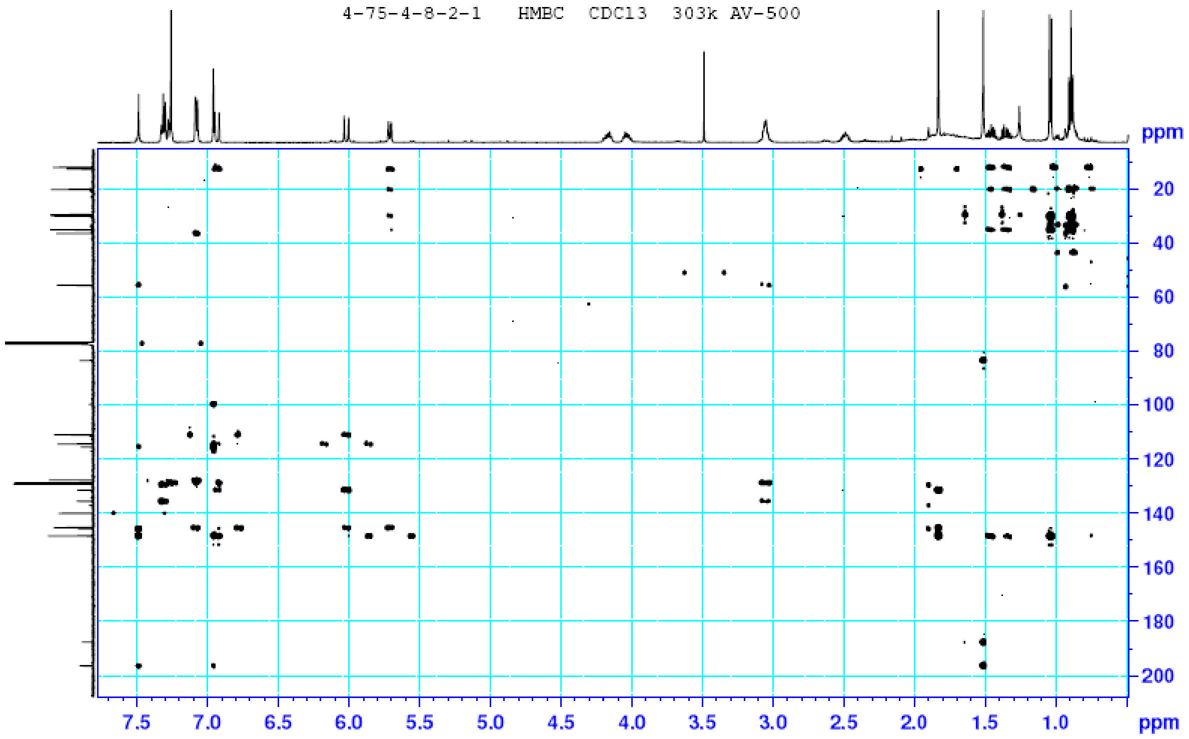

S15. CD spectrum of isochromophilone XI (2) in MeOH.

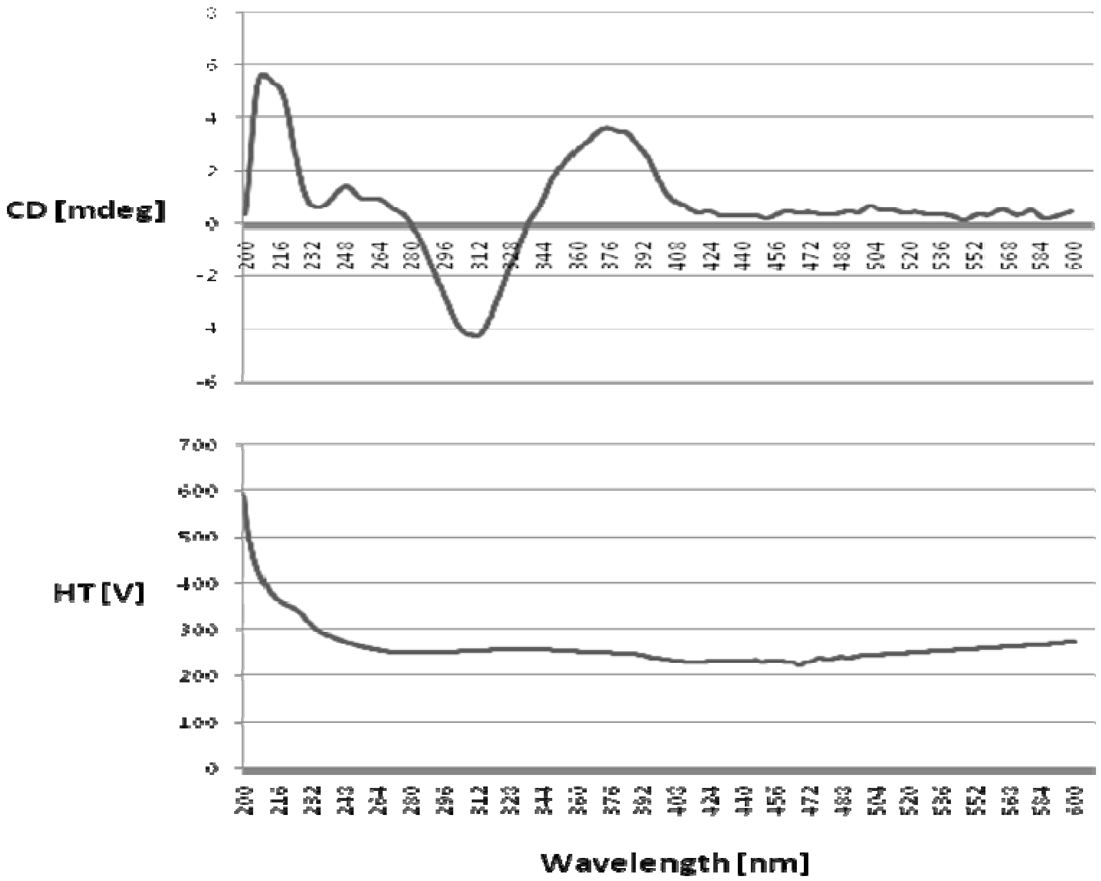

S16. ^{1}H NMR spectrum of isochromophilone XII (3) in $CDCl_3$ (500 MHz).

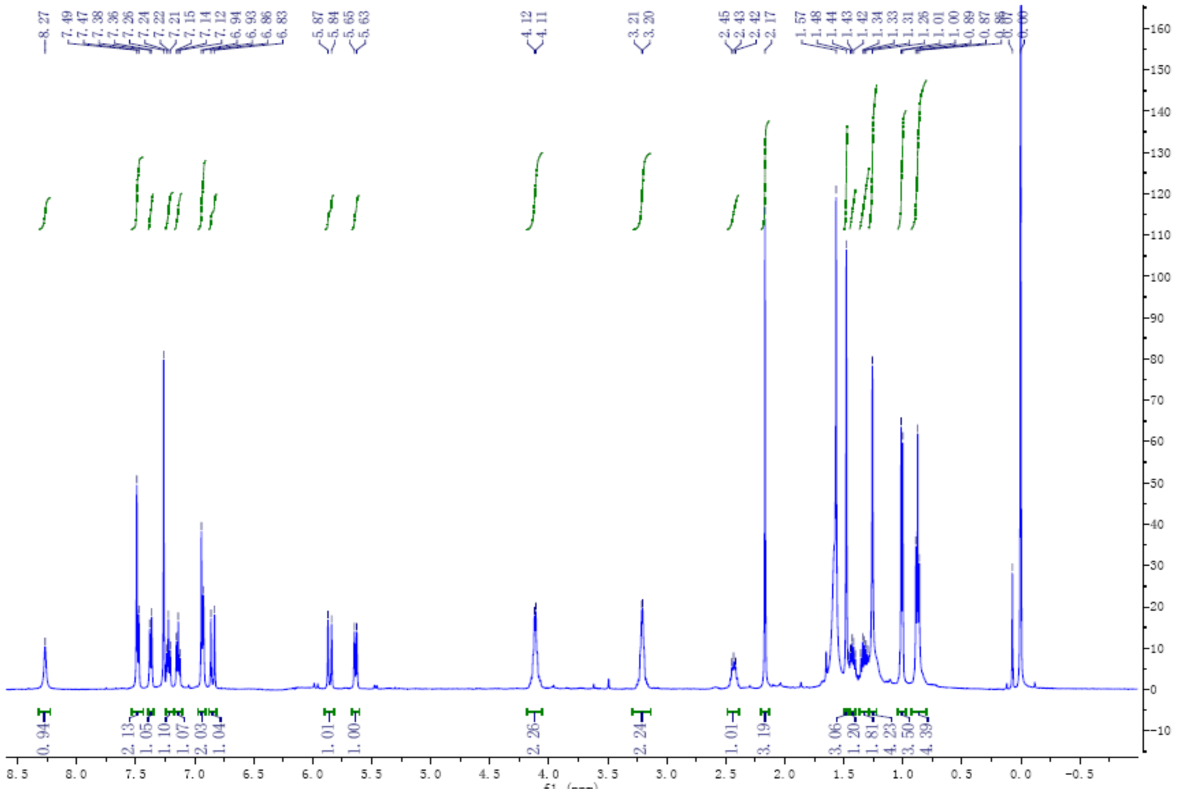

S17. ^{13}C NMR spectrum of isochromophilone XII (3) in $CDCl_3$ (125 MHz).

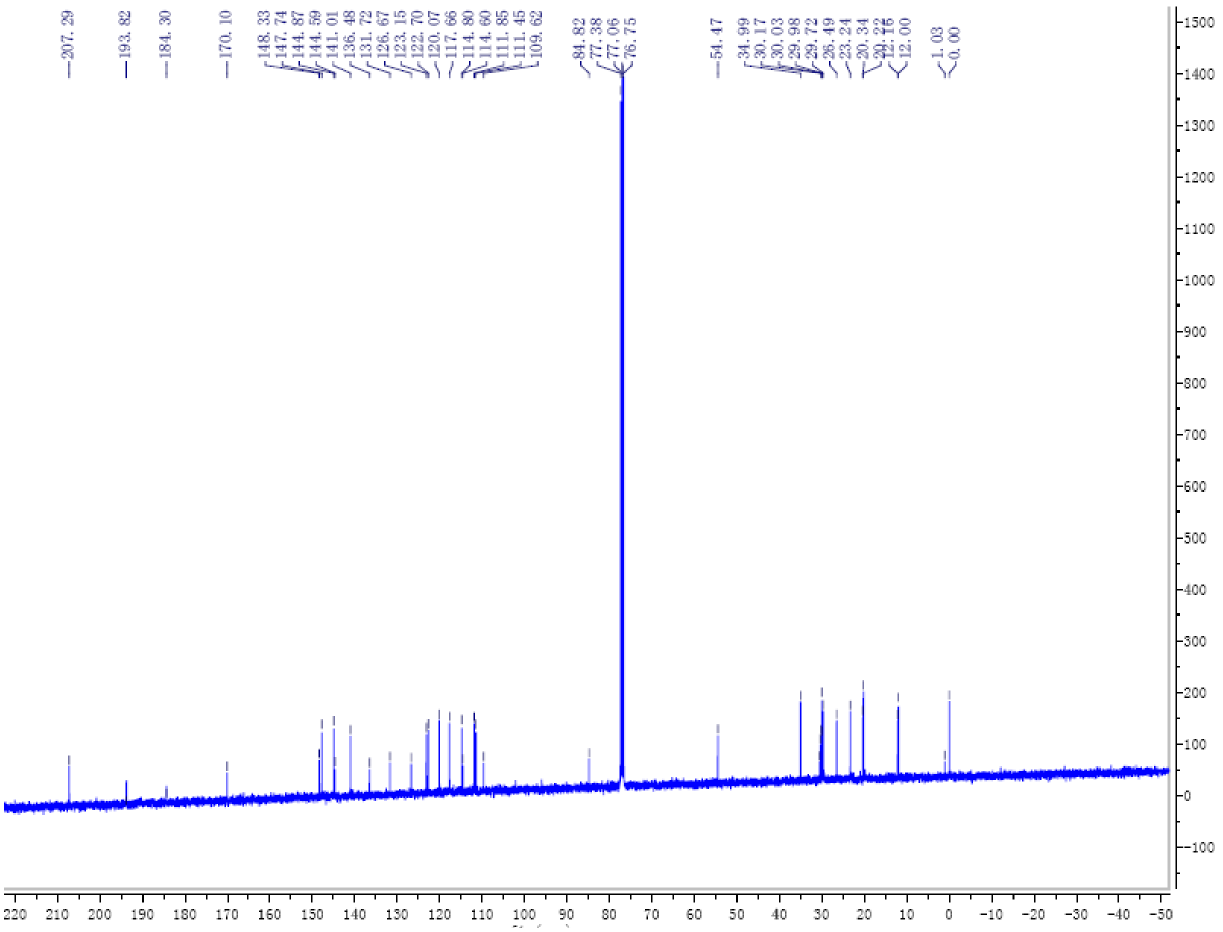

S18. HSQC spectrum of isochromophilone XII (3) in $CDCl_3$ (300 MHz).

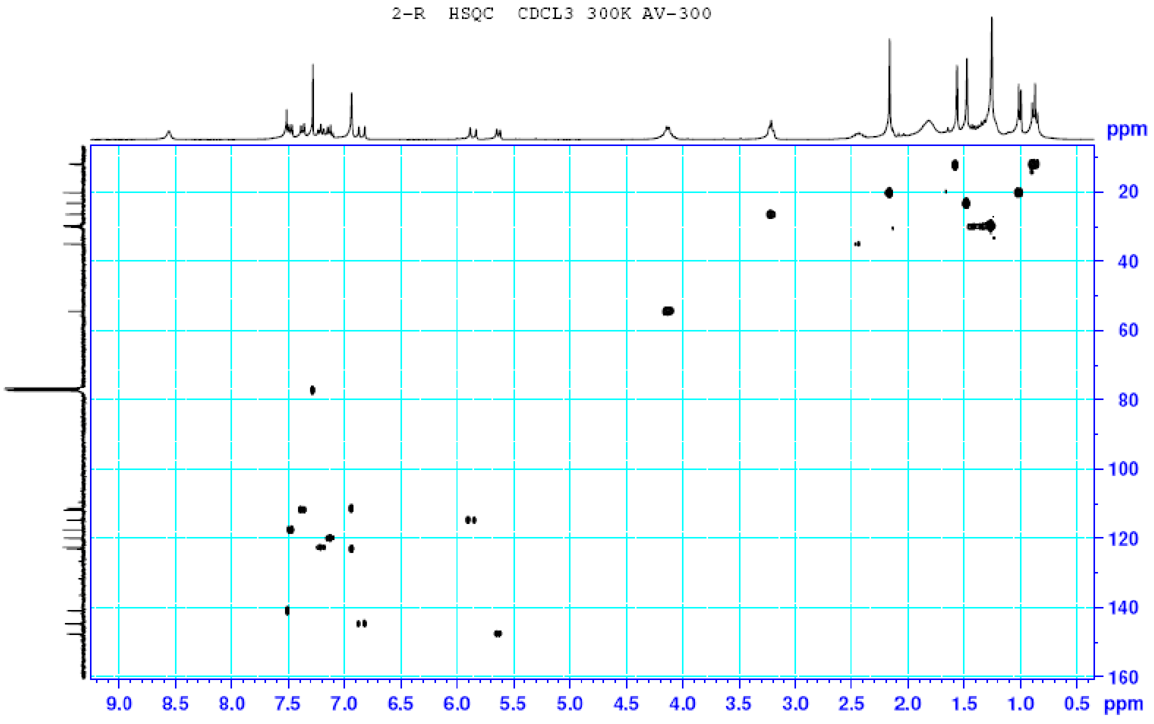

S19. ^{1}H-^{1}H COSY spectrum of isochromophilone XII (3) in $CDCl_3$ (500 MHz).

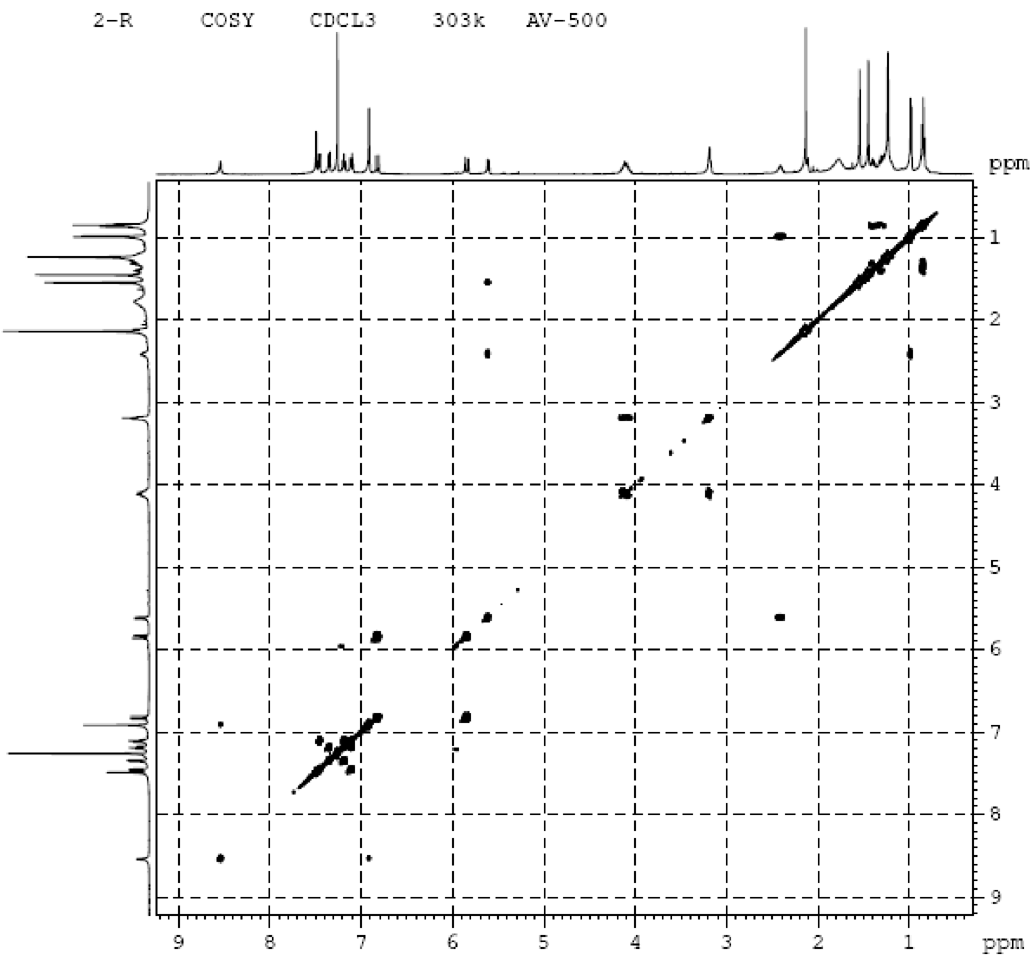

S20. HMBC spectrum of isochromophilone XII (3) in $CDCl_3$ (300 MHz).

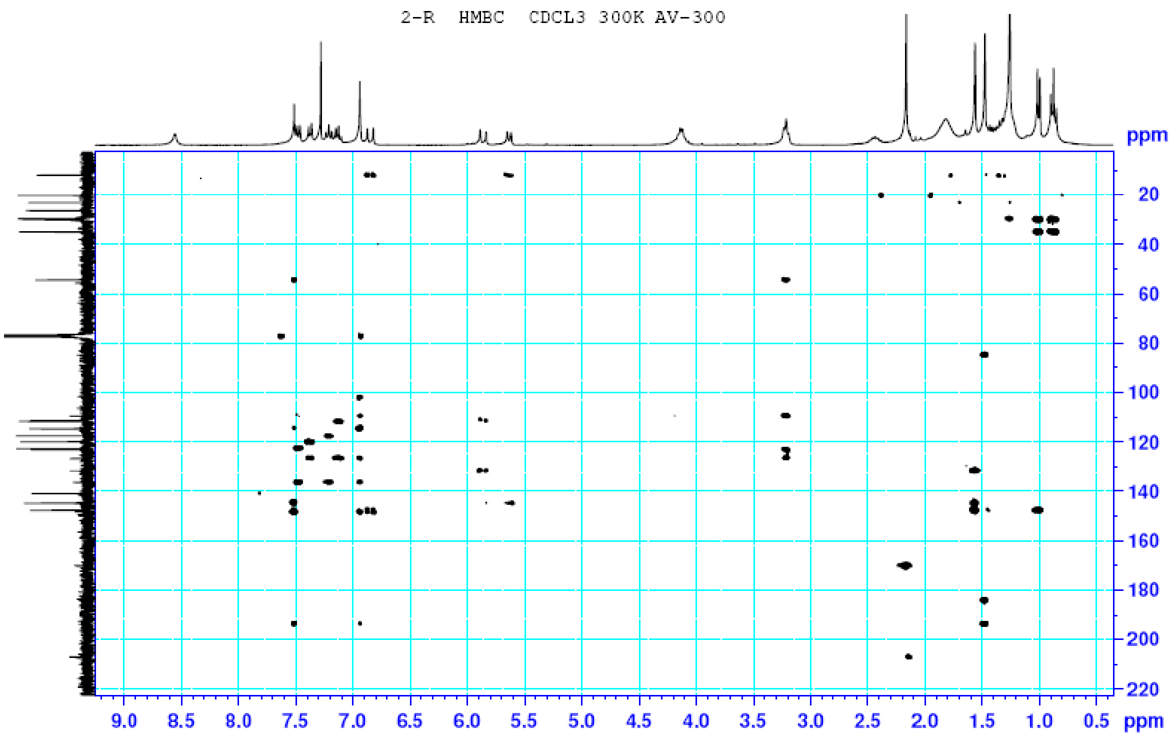

S21. CD spectrum of isochromophilone XII (3) in MeOH.

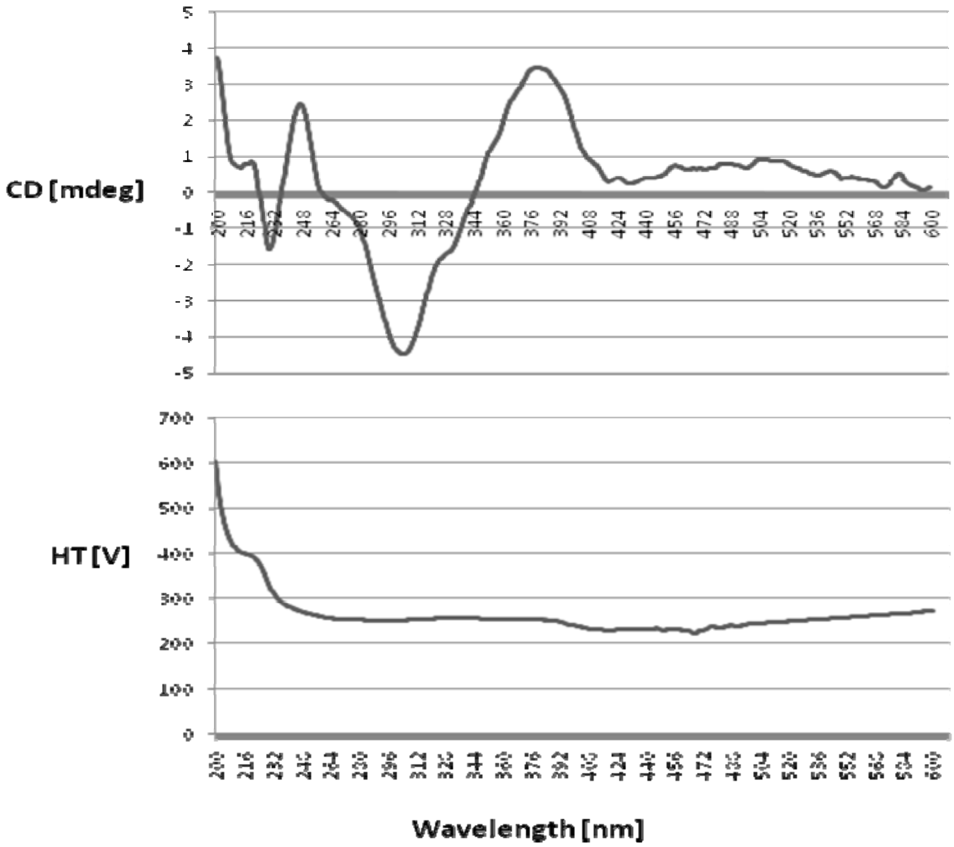

Supplement: Supplementary file 1 — Supplementary material, approximately 819 KB. [file 13659_2012_23_MOESM1_ESM.pdf]
